# Supplementary material for: Determination of melon seed physical parameters and calibration of discrete element simulation parameters
Source: PLoS One. 2024 Jul 15;19(7):e0300516. doi: 10.1371/journal.pone.0300516 (PMC11249231; doi:10.1371/journal.pone.0300516)
Supplement: Attachment — Submitted filename: Comments.docx [file pone.0300516.s006.docx]

**Comments:**

The paper presents a comprehensive study aimed at improving the accuracy of the discrete element model for Hami melon seeds. The calibration of parameters involved a combination of practical experiments and simulation tests, focusing on various physical characteristics such as triaxial size, mass, moisture content, density, Young's modulus, and more. The authors employed rigorous testing methodologies, including the Plackett-Burman test, steepest climbing test, and quadratic regression orthogonal rotation combination test, to identify significant factors influencing the angle of repose.

Here are some specific comments and suggestions for improvement:

1. In the section 2, on intrinsic parameters, discussed the mechanical properties of cantaloupe seeds. How do these properties impact the design and functionality of seed-metering devices?
2. In the section 2.2, on bench testing, discussed the measurement of collision recovery coefficients and static friction coefficients. How do these coefficients affect the interaction of cantaloupe seeds with the sowing machinery, and what implications do they have for real-world applications?
3. In the section 2.2.3, Describe the process and purpose of the angle of repose determination, and why is it considered a critical parameter in discrete element simulation?
4. In section 2.5, The optimization module in Design-Expert 10.0 was employed for solving regression equations. kindly elaborate on the specific criteria and considerations used for optimization, and how these optimized parameters contribute to the accuracy of the discrete element model?
5. In section 2.6, What are the implications of the relative error in the static seed drop rest angle and dynamic rolling stacking inclination for cantaloupe seeds, and how closely did the simulation results match the actual experiments?
6. How did the authors ensure the reliability of the physical tests for determining parameters such as triaxial dimensions, thousand-seed mass, and moisture content?
7. In section 3, line 507, briefly mentioned the levitation speed of cantaloupe seeds. Kindly discuss the relevance of this parameter in the context of cantaloupe seeding and how it aligns with the overall goals of the study?
8. In section 3, What factors influence the relative error in the angle of repose, and how were these factors determined in the simulation experiments?
9. What are the implications of the relative error in the static seed drop rest angle and dynamic rolling stacking inclination for cantaloupe seeds, and how closely did the simulation results match the actual experiments?
10. Provide more references to support the information presented throughout the manuscript.
11. Proofread the manuscript for grammar, punctuation, and formatting errors. Ensure that the text is clear, concise, and free from typos.

Addressing these comments and making the suggested improvements will make the paper more comprehensive, coherent, and reader friendly.
