# Supplementary material for: Determination of melon seed physical parameters and calibration of discrete element simulation parameters
Source: PLoS One. 2024 Jul 15;19(7):e0300516. doi: 10.1371/journal.pone.0300516 (PMC11249231; doi:10.1371/journal.pone.0300516)
Supplement: Attachment — Submitted filename: Reviewer comments.docx [file pone.0300516.s007.docx]

Reviewer’s comments:

2024-Jan-5

| Title | Determination of melon seed physical parameters and calibration of discrete element simulation parameters |
| --- | --- |
| Authors | Wan Yuan, Zhang Bowen, Lai Qinghui, Gong Yan, Yu Qingxu |
| Journal | [PLOS](https://www.letpub.com.cn/index.php?page=journalapp&view=detail&journalid=8695) ONE |

This article describes the simulation of Hami melon seeds and calibration of the micro parameters with DEM. The authors initially obtained the basic parameters of these seeds. Then, the Plackett-Burman (PB) method was employed to screen these parameters. The authors found that the recovery coefficient, static friction coefficient, and rolling friction coefficient of Hami melon seeds significantly affected the angle of repose. The manuscript presents an effective and intriguing study on modeling and calibration of Hami melon seeds, which is suitable for publication in "PLOS ONE." However, to enhance reader comprehension, certain explanations are needed. The following are some highlighted issues.

1. Why was the inclinometer's plate replaced with an ABS plastic plate for testing? If there are some advantages, it would be beneficial to articulate them in the article.
2. How many particles are required to model a seed accurately? Please provide a more detailed explanation.
3. The x and y axes in Figure 8 should be explained, including their units of measurement.
4. There is Chinese text in the P-values section of Table 6. Please make the necessary modifications.
